# Adsorption of Acid Orange Ⅱ with Two Step Modified Sepiolite: Optimization, Adsorption Performance, Kinetics, Thermodynamics and Regeneration

**DOI:** 10.3390/ijerph17051732

**Published:** 2020-03-06

**Authors:** Jian Yu, Wenting He, Bin Liu

**Affiliations:** 1 Department of Water Engineering and Science, College of Civil Engineering, Hunan University, Changsha 410082, China; yujian@hnu.edu.cn (J.Y.); lgc@hnu.com.cn (W.H.); 2 Department of Chemical Engineering, Process Engineering for Sustainable Systems (ProcESS), KU Leuven, Celestijnenlaan 200F, B-3001 Leuven, Belgium

**Keywords:** acid orange II, cetyltrimethylammonium bromide, sepiolite, two steps modification, adsorption

## Abstract

In this study, a two-step modification of sepiolite for adsorption enhancement was investigated. The cetyltrimethylammonium bromide (CTAB) was utilized for the organic modification process after a heat modification. To develop the optimal modification condition, adsorption of Acid Orange II onto modified sepiolite was investigated with respect to heat temperature and adsorbent dosage. The temperature of 200 °C and 100% cation exchange capacity (CEC) was deemed as the optimal condition. The impacts of operation conditions on adsorption procedure, including pH, adsorbent dosage and adsorption duration, were comprehensively discussed. The adsorption of Acid Orange II by sepiolite is in accordance with the quasi-secondary kinetic model. Moreover, the results of intraparticle diffusion indicate that the intraparticle diffusion was the dominant adsorption force in the initial adsorption period. The adsorption process was obeyed with the Langmiur adsorption model. The results from regeneration procedure suggest that the superior regeneration obtained with 0.8 mol/L NaOH concentration.

## 1. Introduction

A dye is an organic compound which stains the substance in an aqueous solution or other medium. The dyestuff is employed with a dispersed state or dissolved in a water solvent to form a solution to dye the material [1,2]. Acid Orange II is a commonly used acidic water-soluble dye and has strong carcinogenicity [3]. It is mainly used in the wool, leather, silk and paper industry. At the same time, it is also an indicator for staining tissue sections. The composition of dye wastewater mainly consists of refractory organic molecules [4]. These organic compounds have the characteristics of being difficult to biodegrade and stable under light, heat and oxidant [5,6,7].

Adsorption is a water remediation process which can utilize porous materials [8]. One or more of the components, by the action of molecular gravitation or chemical bond force, adsorbs the contaminants on the solid surface, thereby achieving the purpose of separation [9]. Commonly used solid adsorbents include activated carbon, zeolite, silica gel, activated alumina, and sepiolite, etc. Moreover, some readily available and cheap materials like desert sand were also employed as adsorbents [10].The adsorption method has the advantage of operation simplicity, treatment effectiveness and operability [11]. It is the most effective method and also the most widely used method for treating wastewater contaminated with substances such as dyes [12,13,14], heavy metal cations [15], or pharmaceuticals [16].

Sepiolite is a hydrous magnesium silicate with a fibrous cross-section [17]. The merits of sepiolite as an adsorbent are low thermal conductivity, high salt resistance, and being non-polluting and environmentally friendly [18]. It has been widely used in various fields due to its special structural properties and low cost. In China, sepiolite presents with two types—thehydrothermal type and clay type. The clay type is mainly distributed in Hunan province [17]. The water in sepiolite mainly exists with three forms—adsorbing water, coordinating water and hydroxyl water, in which the adsorption water enters the sepiolite pores, the coordinating water is mainly bound by Mg^2+^, and the hydroxyl water exists as an OH group [19]. The structure of sepiolite was greatly impacted by the bound water, especially after the sepiolite is heated [20]. The three forms of water are gradually lost, and the structure of sepiolite will change differently at various temperatures. Serna et al. found that the adsorbed water in the sepiolite pores was mainly lost, and the specific surface area of the pores was increased at 25~250 °C, but the sepiolite was folded when the temperature was higher than 300 °C. The possible way of transforming sepiolite is depicted in Equation (1) [21].

(1) $$Mg_{8}Si_{12}O_{30}{\left(OH\right)}_{4}{\left(OH_{2}\right)}_{4}8H_{2}O\overset{\leq 300\;{}^{\circ}C}{\to }\;Mg_{8}Si_{12}O_{30}{\left(OH\right)}_{4}{\left(OH_{2}\right)}_{4}\;\;\;\;\;\;\;\;\;\;\;\;\;\;\;\;\;\overset{\leq 800\;{}^{\circ}C}{\to }\begin{array}{c} \;Mg_{8}Si_{12}O_{30}{\left(OH\right)}_{4}\overset{\leq 900\;{}^{\circ}C}{\to }\;8MgSiO_{3}+4SiO_{2} \end{array}$$

Sepiolite has a large specific surface area and ion exchange capacity, indicating an excellent adsorption property, but its surface is negatively charged and hydrophilic due to surface polarity, which impedes the application of sepiolite as an adsorbent in dye wastewater treatment [22]. Consequently, sepiolite has usually been modified to improve its adsorption property. Not only does a large amount of Mg^2+^ exist in the sepiolite tunnel, but also a small number of cations, such as Ca^2+^ and Na^+^, which endows sepiolite with a certain ion exchange property. The cation exchange capacity (CEC) of sepiolite is basically between 20 and 100 mg/100 g, depending on the origin area and structure, and the cation exchange capacity can be enhanced by organic modification [23,24]. A large number of inorganic ions between the layers is not conducive to its dispersion in the polymer matrix [25]. The surface organic modification can change the high polarity of the surface of sepiolite, transform the sepiolite from hydrophilic to lipophilic, reduce its surface energy, and also increase its interlayer spacing, allowing polymer chains or monomers to enter the interlayer [26,27]. Cetyltrimethylammonium bromide (CTAB) was widely reported in adsorber (e.g., zeolite, bentonite and montmorillonite) modification [28,29,30]. On the other hand, surfactant modification was proven as an efficient method to enhance the adsorption performance of sepiolite [2].

In this study, a two-step modification method was applied for sepiolite adsorption enhancement. The first step was enlarging the adsorption sites by heat modification. The adsorption amounts of Acid Orange II at various temperatures were compared to determine the optimal modification conditions. For the second step, CTAB was employed as the surfactant for organic modification and the primary dosage of CTAB was proposed. The adsorption factors, which impact the adsorption of Acid Orange II by organically modified sepiolite, and which include pH, adsorbent dosage and adsorption duration, were comprehensively discussed. Moreover, the kinetics and thermodynamics were utilized to explain this adsorption mechanism. At last, the regeneration performance of modified sepiolite was reported.

## 2. Method and Materials

### 2.1. Materials

The sepiolite employed in this study was bought from Guangda sepiolite company, China. The cation exchange amount (CEC) of sepiolite was determined to be 101.94 mmol/100 g with the barium chloride-sulfuric acid method [31]. Details of the chemical composition of the sepiolite based on EDX analysis are presented in Table 1. Acid Orange II was purchased from Sigma company and the formula was C_16_H_11_N_2_O_4_SNa. Cetyltrimethylammonium bromide (CTAB) was purchased from Tianjin Bodi Chemical company and its formula was C_16_H_33_ (CH_3_)_3_NBr. Other chemical agents utilized in this work, unless otherwise stated, were analytical grade chemicals. An X-ray diffraction spectrometer (D8-Advance, Burke, Germany) was employed to detect X-ray diffraction spectroscopy (XRD) before and after modification.

### 2.2. Preparation of Organically Modified Sepiolite

The sepiolite was employed for further use after being naturally settled and purified. The sepiolite was dried in a vacuum oven at 120 °C and sieved with a 100 mesh. The purified sepiolite was placed in a muffle furnace and calcined with various ambient temperatures (100, 200, 300, 400 and 500 °C) for 2 h [32]. The water from different components of the sepiolite was lost and the tunnel structure expanded after this process. Then, the sepiolite was added into CTAB saturated solution for complete mixing. The modified sepiolite was dried at 60 °C after the settlement and cleaning procedure. The CTAB equivalents to 20%, 40%, 60%, 80%, 100%, 150% and 200% CEC of sepiolite ore were employed in this study for determining the optimal modification conditions [33].

### 2.3. Adsorption Test

The decolorization ratio and adsorption amount according to the Lambert–Beer law were employed (Equations (2) and (3)) to determine the result of the adsorption procedure [34].

(2) $$\eta \%=\frac{C_{0}-C}{C_{0}}\times 100\%$$

(3) $$q=\frac{\left(C_{0}-C\right)V}{1000m}$$

where *η*% is the decolorization ratio of Acid Orange II, C_0_ and Ce are the initial and final concentration of Acid Orange II, q is the adsorption amount, V is the solution volume and m is the weight of the adsorbent. In order to ensure the accuracy of the test data, each group of experiments was repeated 3 times under the same test conditions and averaged. Unless otherwise mentioned, the adsorption test was conducted with 300 mL dye solution under an oscillation rate of 180 r/min and 25 °C.

In order to study the kinetic characteristics of Acid Orange II removal with sepiolite adsorption, the experimental data were fitted by the quasi-first-order, quasi-secondary and intraparticle diffusion kinetics models. The mathematical expressions of the three kinetic models are listed in Equations (4)–(6), respectively [35].

quasi-first-order model

(4) $$\ln\left(q_{e}-q_{t}\right)\;=\;\ln q_{e}-k_{1}t$$

quasi-secondary model

(5) $$\frac{1}{q_{t}}\;=\;\frac{1}{k_{2}q_{e}^{2}}+\frac{1}{q_{e}}t$$

intraparticle diffusion model

(6) $$q_{t}\;=\;k_{p}t^{0.5}+c$$

where q_e_ is the amount of Acid Orange II adsorbed by the unit mass adsorbent after equilibrium, q_t_ is the amount of Acid Orange II adsorbed by the unit mass adsorbent at the moment t, k_1_ is the adsorption rate constant of the quasi-first-order reaction kinetic equation, k_2_ is the adsorption rate constant of the quasi-second-order reaction kinetic equation, k_p_ is the adsorption rate constant of the intramolecular diffusion kinetic equation, and c is the intercept, representing the thickness of the boundary layer.

In order to study the thermodynamics characteristics of Acid Orange II removal with sepiolite adsorption, the experimental data were fitted by the Langmuir linear fitting and Freundlich linear fitting models. The mathematical expressions of the thermodynamics models are listed in Equations (7) and (8), respectively [36].

Langmuir linear fitting model

(7) $$\frac{c_{e}}{q_{e}}\;=\;\frac{1}{q_{max}K_{L}}+\frac{c_{e}}{q_{max}}\;\;and\;\;R_{L}\;=\;\frac{1}{1+K_{L}c_{0}}$$

Freundlich linear fitting model

(8) $$lgq_{e}\;=\;lgK_{F}+\frac{1}{n}lgC_{e}$$

where q_e_ is the balanced amount of adsorption, q_max_ is the saturated adsorption capacity of a single layer, c_e_ is the balanced mass concentration of the dye, K_L_ is the adsorption equilibrium constant in Langmuir linear fitting model, R_L_ represents the adsorption procedure, and K_F_ and n are the adsorption constants.

### 2.4. Alkali Regeneration Test

During the regeneration test, the adsorbent which had reached the adsorption equilibrium at the condition of 2 g/L modified sepiolite and 200 mg/L Acid Orange II was immersed into 50 mL NaOH solution after 70 °C drying. The mixture was shaken with 180 r/min oscillation speed at a normal temperature for a certain period of time, and then the adsorbent was filtered out and dried at 75 °C to obtain a regenerated organic adsorbent. Then, 2 g/L of regenerated adsorbent was dosed into 200 mg/L Acid Orange II solution (50 mL) for 2 h at the condition of 180 r/min oscillation rate to determine the adsorption regeneration ratio. The effects of different sodium hydroxide concentrations, reaction durations and reproduction times on regeneration performance were investigated.

## 3. Result and Discussion

### 3.1. Effect of Modification Condition on the Adsorption Performance

**Figure 1:**
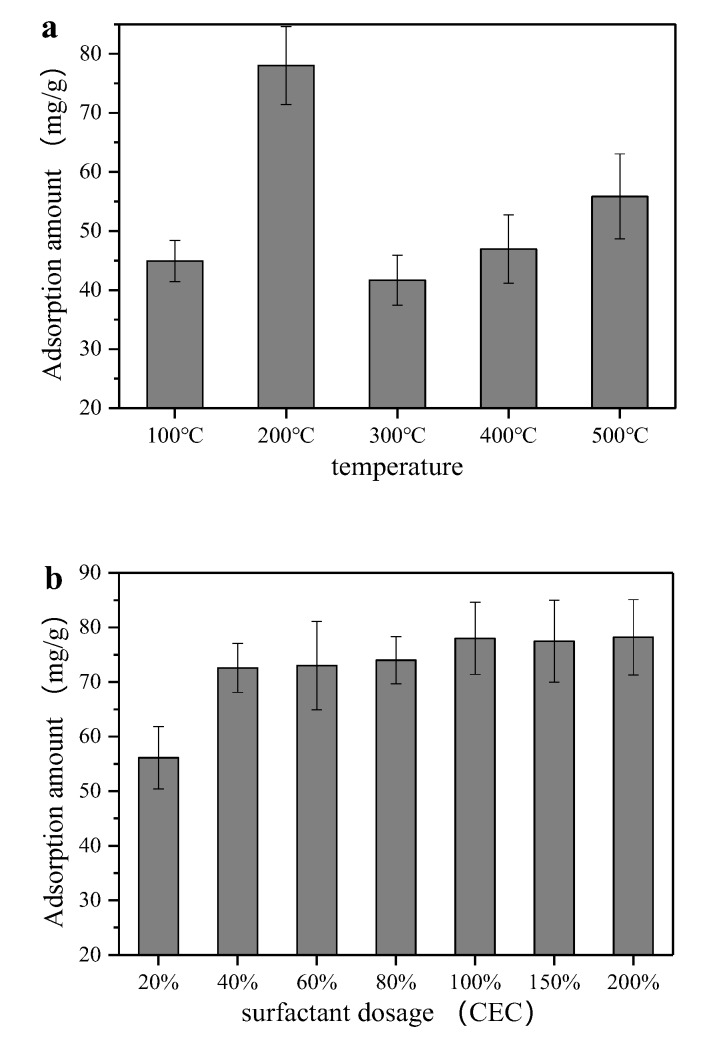
Effect of temperature of heat process (**a**) and CTAB concentration (**b**) on the sepiolite modification performance. To evaluate the adsorption amount, 2 g/L modified sepiolite was dosed to 50 mL Acid Orange II solution (200 mg/L) with pH value of 6.5 and then oscillated in a 180 r/min oscillation box for 4 h under constant temperature (25 °C). In (**a**), the 100% CEC CTAB-modified sepiolite was utilized; In (**b**), the modified sepiolite after 200 °C heat modification process was utilized.

As shown in Figure 1a, in the temperature range of 100 to 200 °C, the adsorption amount of Acid Orange II was enhanced with the increase in temperature, and the adsorption amount at 200 °C was twice the amount at 100 °C. The sepiolite lost more moisture at 200 °C, and hence the adsorption resistance of the water film to the pollutants reduced. However, when the calcination exceeded 200 °C, the adsorption amount declined. The possible reason for the decrease in the adsorption amount was that the structure of sepiolite was destroyed and the crystal structure was deformed. Although the adsorption amount slightly increased when the temperature exceeded 300 °C, the adsorption amount after treatment at 500 °C was much lower than that at 200 °C [21]. Figure 1b shows that the adsorption amount improved with the increase in CTAB dosage. When the concentration of CTAB reached 100% CEC, the adsorption amount increased from 56.1 to 78 mg/g. The adsorption amount was stabilized when the surfactant dosage continued to increase. The surface charge of sepiolite tends to be more positive when introducing the CTAB which could improve the adsorption of negatively charged Acid Orange II. The more organophilic surface of modified sepiolite could also heighten the adsorption amount. However, the excess cetyltrimethylammonium bromide would form a “dual layer” on the sepiolite surface by van der Waals force when CTAB is over 100% CEC, which could lead to the outward hydrophilic group forming the outer “dual layer” [37].

**Figure 2:**
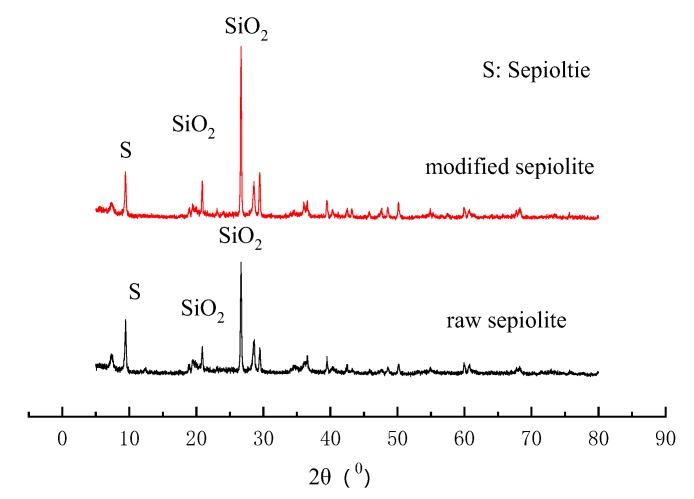
X-ray diffraction spectra of original sepiolite and modified sepiolite.

Figure 2 shows the X-ray diffraction analysis spectra of sepiolite before and after CTAB modification. When comparing the X-ray diffraction of modified sepiolite and raw sepiolite, the diffraction peak intensity of sepiolite and quartz sand was slightly reduced and no significant change in its characteristic peak position was observed. This indicates that although the surfactant CTAB modifies the sepiolite surface, it does not change the crystal structure of sepiolite. The functional modification of CTAB mainly occurrs on the surface of the raw sepiolite, thereby maintaining the original sepiolite structure. This is also in accordance with previous research [2,17].

**Figure 3:**
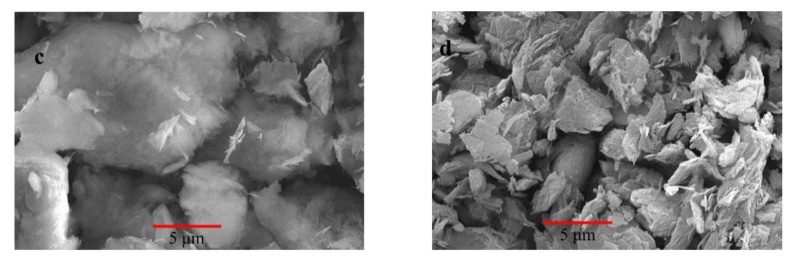
SEM image of original sepiolite (**a**) and modified sepiolite (**b**) in the literature [2].

Figure 3 shows morphology images from our previous work, which used sodium dodecylbenzene sulfonate (SDBS) to modify sepiolite [2]. It confirms that the original crystal structure of sepiolite was kept. A loose structure and smooth interval was obtained after the SDBS modification. In this study, the CTAB modification was utilized, and, based on the previous work and X-ray diffraction spectra, the CTAB probably modified the surface of sepiolite with the dominated mechanism of electrostatic attraction and ion exchange.

### 3.2. Effect of the Operation Condition on Adsorption Performance

**Figure 4:**
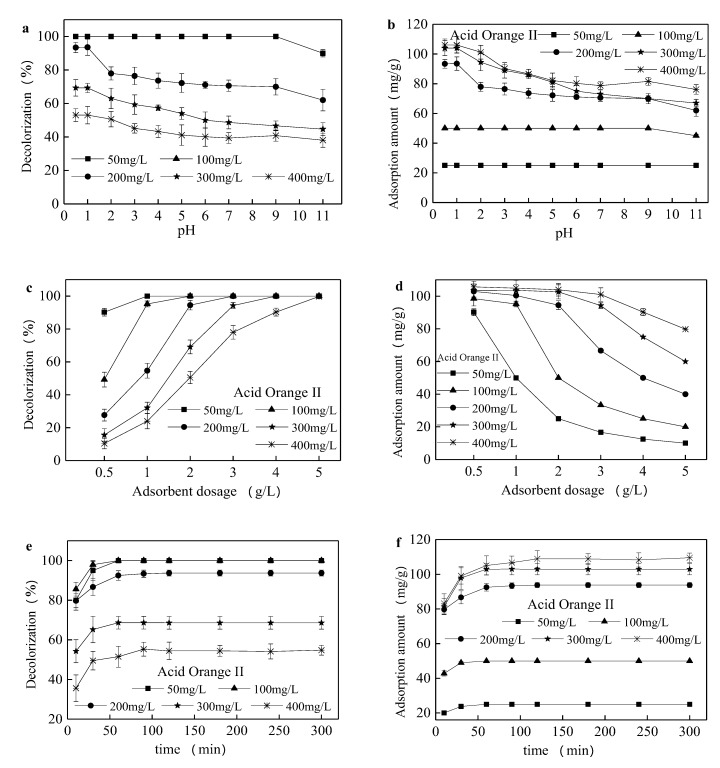
Effect of pH, adsorbent dosage and adsorption duration on the Acid Orange II adsorption performance. In (**a**,**b**), the adsorption test was conducted with 2 g/L modified sepiolite for 120 min under an oscillation rate of 180 r/min and 25 °C; in (**c**,**d**), the adsorption test was conducted for 120 min under initial pH value of 1, an oscillation rate of 180 r/min and 25 °C; in (**e**,**f**), the adsorption test was conducted with 2 g/L modified sepiolite under an oscillation rate of 180 r/min, an initial pH value of 1 and 25 °C.

In spite of a much higher adsorption capacity obtained through the organic modification, the adsorption performance of anionic dye was also restricted by many factors, such as pH, adsorbent dosage and adsorption duration [38]. As can be seen from Figure 4a,b, the pH value of the solution had a great influence on the adsorption effect, especially with higher adsorbate concentration. When the initial concentration reached 200 mg/L, the adsorption amount declined from 93.78 to 64.2 mg/g with the increase in pH. Since the positively charged adsorption site of the adsorbent surface increased under acidic conditions, the electrostatic attraction to the negatively charged anionic dye could be improved. The positive charge on the adsorbent surface reduced with the pH increase and gradually exhibited a negative charge, which could lead to repulsion between the adsorbent and the anionic dye. Figure 4c,d show that the decolorization rate of organically modified sepiolite to Acid Orange II increased rapidly, but the adsorption amount decreased when the dosage increased from 0.5 to 5 g/L. When the dosage of adsorbent was fixed, the modified sepiolite had a large amount of superfluous silicic hydroxyl groups and obvious pore effects with a small amount of Acid Orange II. However, when the Acid Orange II was excessive, the sepiolite quickly reached saturation and the surface free energy gradually decreased.

Adsorption duration is an important factor in studying adsorption performance and kinetics. As shown in Figure 4e,f, the entire adsorption process can be divided into two phases. In the first 60 min, the Acid Orange II is rapidly adsorbed, and over 90% of the Acid Orange II is adsorbed after 300 min. At the second stage, the adsorption rate is mitigated, and the adsorption basically reaches equilibrium after 120 min. The equilibrium adsorption amounts with a sepiolite dosage from 0.5 to 5 g/L were 25, 50, 93.78, 102.91, and 108.89 mg/g, respectively.

### 3.3. Adsorption Kinetics and Adsorption Thermodynamics

**Figure 5:**
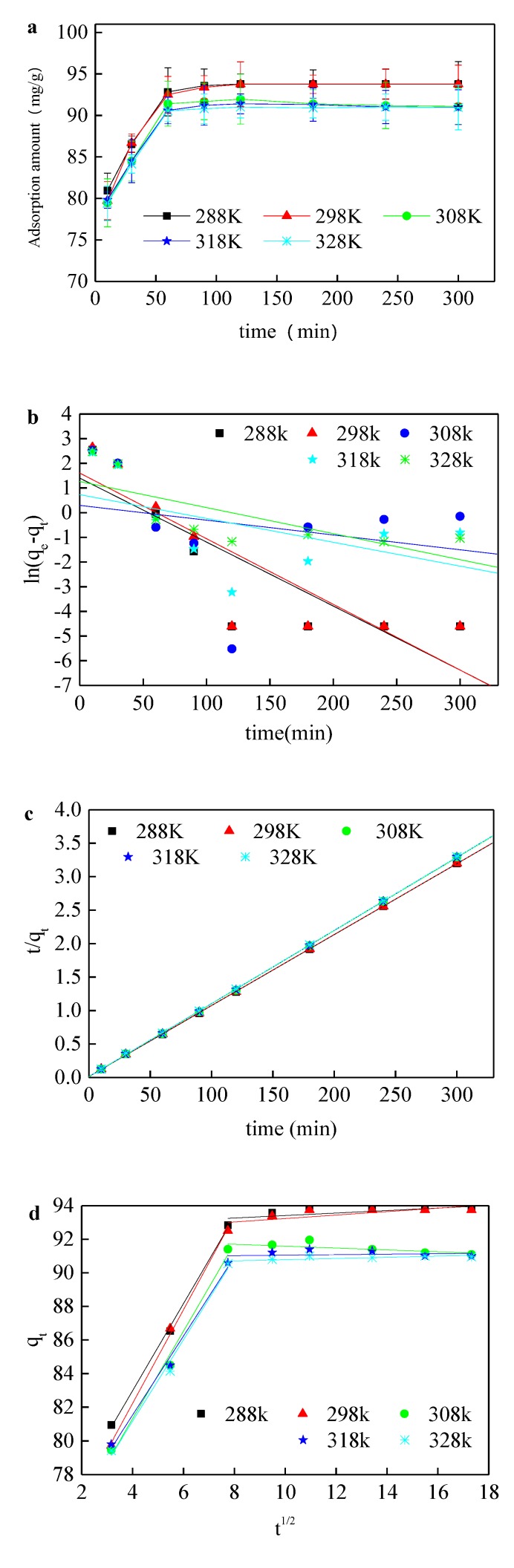
(**a**) Effect of adsorption duration on the adsorption amount at various temperatures; (**b**) fitting of experimental data with the quasi-first-order reaction kinetics model at various temperatures; (**c**) fitting of experimental data with the quasi-second-order reaction kinetics model at various temperatures; (**d**) fitting of experimental data with the intraparticle diffusion model at various temperatures. The adsorption test was conducted with 200 mg/L Acid Orange II and 2 g/L modified sepiolite under an oscillation rate of 180 r/min and an initial pH value of 1.

Figure 5a shows the variation of adsorption amount with temperature. Similarly to Figure 5f, the whole adsorption process was divided into two stages, namely the rapidly adsorption stage at 0–60 min and the plain stage after 60 min. The adsorption amount reached the maximum at 120 min. The adsorption reaches equilibrium irrespective of applied temperature. The adsorption amount declined from 93.78 to 90.95 mg with the increase in temperature from 288 to 328 K, indicating that the adsorption amount decreased with the rise in temperature and the whole adsorption process is in an exothermic state, although the temperature has a limited effect on the adsorption. When the temperature is over 298 K, the adsorption amount of the adsorbent on Acid Orange II decreased slightly after the equilibrium time, which indicates that the desorption happened at a high temperature. To investigate the adsorption kinetics, the quasi-first-order reaction kinetics model, quasi-second-order reaction kinetics model at various temperatures, and the intraparticle diffusion model were employed. The adsorption of Acid Orange II by organic sepiolite is consistent with the quasi-secondary reaction kinetics model rather than the quasi-first reaction kinetics model at different temperatures [33]. Table 2 shows that the correlation coefficients of the quasi-secondary reaction kinetics model were over 0.9999, and the correlation coefficients of quasi-first-order reaction kinetics were between 0.2726 and 0.7520. When the temperature raised from 288 to 328 K, the quasi-secondary reaction rate constant k_2_ was increased from 6.7 × 10^−3^ to 1.27 × 10^−2^. The migration speed of Acid Orange II accelerated, and the viscosity of the liquid decreased with the rise in temperature, which leads to the reduction in mass transfer resistance and the enhancement of adsorption rate. Adsorption kinetics are generally controlled by a variety of mechanisms, where the diffusion mechanism plays a decisive role. The initial curve segment mainly includes rapid external diffusion, boundary layer diffusion and surface diffusion; the plain segment is mainly a gentle adsorption segment. The adsorption site reduced, and the internal diffusion of the particle was alleviated in this stage.

As shown, the correlation coefficient of the intraparticle diffusion model reached 0.99 in the initial stage. Then, the adsorption rate of the second stage was mitigated and the adsorption basically reached equilibrium. Moreover, the correlation coefficients of the intraparticle diffusion model were below 0.57 in the second stage. This indicates that the intraparticle diffusion was the dominating adsorption force in the first stage, and this effect gradually diminished when approaching the equilibrium time. The fitting line without passing through the origin point indicates that the intraparticle diffusion is not the only mechanism for controlling the adsorption process.

**Figure 6:**
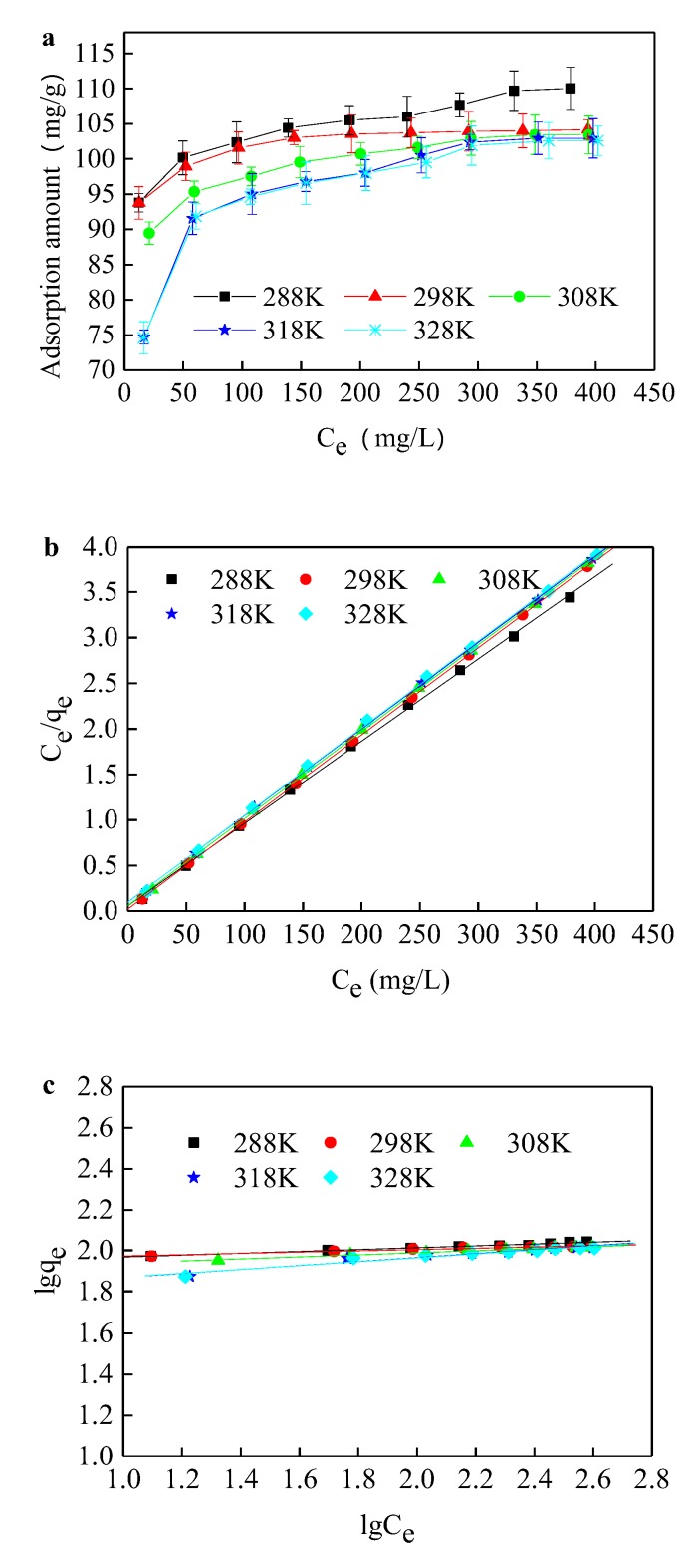
(**a**) Adsorption isotherm at various temperatures; (**b**) Langmuir linear fitting of experimental data at various temperatures; (**c**) Freundlich linear fitting of experimental data at various temperatures. To ensure the adsorbed saturation, the adsorption test was conducted with 200 mg/L Acid Orange II and 2 g/L modified sepiolite under an oscillation rate of 180 r/min and an initial pH value of 1 for at least 10 h at a certain temperature in a constant temperature oscillating box.

The adsorption isotherm can well reflect the relationship between the equilibrium adsorption amount (q_e_) and the equilibrium concentration (C_e_), which plays an important role in explaining the adsorption mechanism (Figure 6). The Langmuir and Freundlich equations have commonly been used for describing isotherms [39]. The Langmuir equation assumes that the adsorption is monolayer adsorption. Each molecule is adsorbed by only a specific number of adsorption sites on the surface of the adsorbent, and no adsorption mass transfer motion happens. The Freundlich adsorption equation is an empirical equation for non-ideal adsorption of non-uniform sorbent surfaces. As shown in Table 3, the fitting result of the Langmuir equation is better than that of the Freundlich equation, indicating that the adsorption of Acid Orange II by modified sepiolite is consistent with the characteristics of single-layer adsorption. The equilibrium constant, K_L_, decreased with an increase in temperature, which confirmed that the adsorption process is an exothermic reaction. The R_L_ values at different temperatures were below 1, indicating that the adsorption of Acid Orange II is preferential adsorption. K_F_ and n are Freundlich adsorption constants. The adsorption is linear when n = 1, the adsorption is difficult to carry out when 1 < n < 2, and the adsorption is easy when n > 2. From the result of Freundlich equation fitting, n > 2, indicating that the adsorption process is easy to carry out. Table 3 shows that the adsorption linear fitting of the Langmuir model was better than the Freundlich model, which suggests that the adsorption of CTAB-modified sepiolite was in line with the Langmuir fitting model and the maximum adsorption capacity for Acid Orange II was 110.05 mg/g. Table 4 presents a comparison of maximum adsorption capacity between the CTAB-modified sepiolite and other reported adsorbent, which suggest that the adsorption performance of CTAB-modified sepiolite was excellent, especially considering the adsorbent cost [28,39,40].

Thermodynamics mainly involves three main parameters, namely enthalpy change, ΔH, entropy, ΔS, and Gibbs free energy change, ΔG. The relationship between these three parameters is presented in Equations (9) and (10) [39].

**Figure 7:**
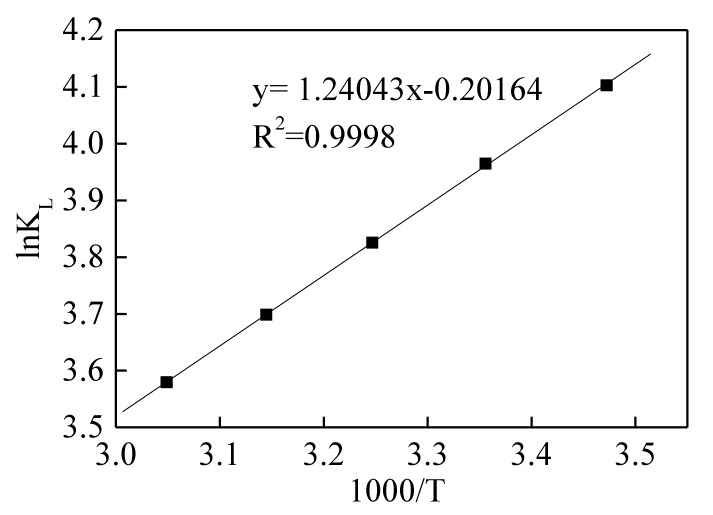
Correlations between 1000/T and ln K_L_.

The adsorption process is an exothermic reaction when ΔH < 0, and, vice versa, it is an endothermic reaction; the reaction can proceed spontaneously when ΔG < 0—otherwise, it is balanced or not spontaneous; the ΔS was always below 0, since the system tends towards disorder and increase chaos. The ΔS and ΔH presented in Table 5 can be calculated according to the slope and intercept obtained by linear fitting in Figure 7.

(9) $$\Delta G\;=\;\Delta H-T\Delta S\;=\;-\mathrm{RT}lnK_{L}$$

(10) $$lnK_{L}\;=\;\frac{\Delta S\;}{R}-\frac{\Delta H}{RT}$$

where T is the absolute temperature, K_L_ is the Langmuir equilibrium constant, R is the ideal gas constant, (8.314 J/mol·K).

### 3.4. Regeneration of Organic Sepiolite Adsorbent

The property of the adsorbent not only depends on the high adsorption capacity and the removal rate, but also the regeneration performance. The adsorbate desorbed from the adsorbent is the principle of adsorbent regeneration and is usually achieved by changing the chemical properties of the adsorbate or using more affinity solvent for extraction [41]. The regeneration rate firstly increased and then decreased with the increase in NaOH concentration. At 0.8 mol/L, the decolorization ratio reached 62.23%. The result indicates that the adsorption was dominated by ion exchange. On the one hand, OH^−^ exchanged with the anion of Acid Orange II, and the dye was replaced from modified sepiolite. On the other hand, the surface of sepiolite was negatively charged in the strong alkali environment, which destroyed the adsorption equilibrium and resulted in the desorption of Acid Orange II. Table 6 shows that the regeneration efficiency was enhanced when the regeneration duration increased to 2 h, but no significant effect on rejuvenation was observed when regeneration duration over 2 h.

## 4. Conclusions

In this study, the optimization of sepiolite modification with CTAB, adsorption performance, kinetics, thermodynamics analysis and adsorbent regeneration were comprehensively investigated. The conclusions are listed as follows:

(1) The optimal preparation conditions for modified sepiolite are listed as follows: the calcination temperature was 200 °C and the amount of CTAB was equal to the sepiolite cation exchange capacity. The thermal modification does not effectively enlarge the pores at low temperature, while the internal pores collapse at high temperatures. The proper amount of CTAB could form an organic hydrophobic layer and change the negative charge of the surface of the sepiolite by ion exchange.
(2) The decolorization rate of acid orange II by organic sepiolite decreased with the increase in pH. With the increase in adsorbent dosage, the decolorization rate of acid orange II increased, while the adsorption amount declined. In the first 60 min, the adsorption amount can reach 90% of the total capacity, and the adsorption basically reaches equilibrium after 120 min.
(3) The adsorption of Acid Orange II by modified sepiolite was obeyed with quasi-secondary reaction kinetics and the Langmuir equation at different temperatures. The intraparticle diffusion was the dominant adsorption force in the first 60 min and gradually diminished.
(4) The optimal regeneration condition is 0.8 mol/L NaOH and 2 h regeneration duration. Over 50% decolorization rate can be achieved within three regeneration times.

## Figures and Tables

**Table 1:** Chemical Composition of the Sepiolite.

Composition	SiO_2_	MgO	CaO	Al_2_O_3_	K_2_O	Fe_2_O_3_	TiO_2_
Percentage (%)	69.41	17.93	4.18	5.65	0.57	1.91	0.35

**Table 2:** Effect of Temperature on the Adsorption Kinetics Model. The Adsorption Test was Conducted with 200 mg/L Acid Orange II and 2 g/L Modified Sepiolite under an Oscillation Rate of 180 r/min and an Initial pH Value of 1.

**Temperature (K)**	**Quasi-First-Order Reaction Kinetics Model**				**Quasi-Secondary Reaction Kinetics Model**		
	***q_e_*** **(mg/g)**	***q*_1_*_e_*** **(mg/g)**	***k*_1_** **(1/min)**	***R*_1_^2^**	***q*_2_*_e_*** **(mg/g)**	***k*_2_** **(1/min)**	***R*_2_^2^**
288	93.91	98.15	2.59 × 10^−2^	0.7398	94.43	6.7 × 10^−3^	0.9999
298	93.78	97.76	2.66 × 10^−2^	0.7520	94.07	7.3 × 10^−3^	1.0000
308	91.96	179.38	5.99 × 10^−3^	0.0647	91.58	8.4 × 10^−3^	0.9999
318	91.30	133.24	9.66 × 10^−3^	0.2726	91.49	1.01 × 10^−2^	0.9999
328	91.00	91.30	1.06 × 10^−1^	0.5504	91.41	1.27 × 10^−2^	0.9999
**Temperature (K)**	**Intraparticle Diffusion Model**						
	***q_e_*** **(mg/g)**	$k_{1p}$ **(1/min)**	C	***R_*1*p_*** **^2^**	$k_{2p}$ **(1/min)**	**C**	***R_*2*p_*^2^**
288	93.91	2.59	72.6	0.9984	0.074	6.7 × 10^−3^	0.5175
298	93.78	2.78	72.4	0.9979	0.1029	7.3 × 10^−3^	0.5627
308	91.96	2.60	70.9	0.9909	0.056	8.4 × 10^−3^	0.4189
318	91.30	2.35	72.1	0.9932	0.015	1.01 × 10^−2^	0.0351
328	91.00	2.43	71.4	0.9914	0.035	1.27 × 10^−2^	0.5526

**Table 3:** Langmuir Linear Fitting and Freundlich Linear Fitting Results of Modified Sepiolite Adsorption.

Temperature (K)	Langmuir				Freundlich		
	q_max_ (mg/g)	K_L_ (10^−2^ L/mg)	R_L_	R_1_^2^	n	K_F_(10^−2^ L/mg)	R_2_^2^
288	110.05	1.73	0.028	0.9994	22.08	83.56	0.9851
298	104.17	1.50	0.032	0.9997	32.29	87.41	0.9559
308	103.42	1.30	0.037	0.9998	20.07	77.29	0.9921
318	102.93	1.16	0.041	0.9995	10.39	59.14	0.9406
328	102.58	1.02	0.047	0.9995	10.64	59.58	0.9394

**Table 4:** Maximum Adsorption Capacity Comparison of Surfactant Adsorbent.

Adsorbent	Surfactant	qmax (mg/g)	Reference
Montmorillonite	Sodium dodecyl sulfate	48.3	Sánchez-Martín et al. [28]
Illite	Sodium dodecyl sulfate	90.1	
Muscovite	Sodium dodecyl sulfate	24.8	
Kaolinite	Sodium dodecyl sulfate	87	
Sepiolite	Sodium dodecyl sulfate	66.2	
Palygorskite	Sodium dodecyl sulfate	44.2	
Alumina	Sodium dodecyl benzenesulfonate	19.8	
Attapulgite	Polyethersulfone	102.04	Yu et al. [28]
Zeolite	Cetyl trimethylammonium bromide	30.7	Taffarel et al. [28]
Silkworm exuviae	Hexadecyltrimethylammonium bromide	87.03	Chen et al. [28]
Sepiolite	Cetyltrimethylammonium Bromide	110.05	This work

**Table 5:** Adsorption Thermodynamic Parameters at Different Temperatures.

Temperature (K)	ΔH(kJ/mol)	ΔG(kJ/mol)	ΔS(J/mol⋅K)
318	−10.31	−9.82	−1.676
328		−9.82	
338		−9.80	
348		−9.76	

**Table 6:** Effect of NaOH concentration, regeneration duration and numbers of regenerations on decolorization ratio. To investigate the NaOH concentration, 8 h regeneration duration was employed; to investigate the regeneration duration, 0.8 mol/L NaOH was employed; to investigate the number of regenerations, 8 h regeneration duration and 0.8 mol/L NaOH was employed.

NaOH Concentration (mol/L)	Decolorization Ratio (%)	Regeneration Duration (h)	Decolorization Ratio (%)	Numbers of Regeneration	Decolorization Ratio (%)
0	18.26	0.5	21.67	0	93.4
0.1	50.65	1	51.20	1	62.03
0.4	55.46	2	62.03	2	55.61
0.8	62.23	4	62.23	3	50.72
1	58.10	8	62.22	4	40.11
